# A comparative study of phyllostachys edulis and its dwarf variant phyllostachys edulis ‘Tubaeformis’ at the anatomical, transcriptomic, and DNA methylation levels

**DOI:** 10.3389/fpls.2026.1747179

**Published:** 2026-02-04

**Authors:** Qiu Zhenhua, Sun Yuanyuan, Lin Shuyan, Liu Xinyao, Li Long

**Affiliations:** 1 National Key Laboratory for the Development and Utilization of Forest Food Resources, Nanjing Forestry University, Nanjing, China; 2 Bamboo Research Institute, Nanjing Forestry University, Nanjing, China; 3 College of Life Sciences, Nanjing Forestry University, Nanjing, China; 4 Laboratory of Pathogen Research, West China Hospital, Sichuan University, Chengdu, China

**Keywords:** DNA methylation, EXPA, Expansin-like A, GRF (growth-regulating factor), *GRF10* (Growth-regulating factor 10)

## Abstract

Internode length is an important trait of bamboo and a key indicator affecting the processing and utilization of bamboo materials. Shengyin bamboo is a dwarf variant of *Phyllostachys edulis* (Moso bamboo) with abnormally shortened internodes, yet its dwarfing mechanism has not been clarified. In this study, we adopted the method of Whole-Genome Bisulfite Sequencing (WGBS) for DNA methylation combined with RNA Sequencing (RNA-seq) to explore the key causes of dwarfism in Shengyin bamboo. Observations via paraffin sections and scanning electron microscopy (SEM) indicate that abnormal cell division and elongation in internodes are the key causes of dwarfism in Shengyin bamboo. Cell division-related genes such as GRF (Growth-regulating factor) and Cyclin are highly expressed during the cell division stage (early growth stage) of Moso bamboo internodes, while genes associated with cell elongation (Expansin-like A, EXPA) are highly expressed during the cell elongation stage (late growth stage) of Moso bamboo internodes. DNA methylation levels exhibit significant differences between Moso bamboo and Shengyin bamboo. Specifically, the DNA methylation level of Moso bamboo at the late stage of internode elongation is higher than that at the early stage, and this difference is significantly greater than the variation observed between the late and early stages of internode elongation in Shengyin bamboo. The expression of most genes shows a negative correlation with promoter methylation levels, indicating that methylation levels inhibit gene expression. Based on transcriptome data, *GRF10*, a gene potentially highly expressed in the early stage of internode growth of Moso bamboo under DNA methylation regulation, was screened out. Genetic transformation of rice showed that GRF10 can promote the growth and development of rice internode cells. In summary, under the regulation of DNA methylation, the expression of genes involved in internode cell division and elongation is inhibited, leading to fewer longitudinal cell lengths and cell numbers in the internodes of Shengyin bamboo compared to Moso bamboo, ultimately resulting in the shortened internodes of Shengyin bamboo.

## Introduction

Moso bamboo (*Phyllostachys edulis*) belongs to the Bambusoideae subfamily of the Poaceae family (formerly known as Gramineae). It is one of the plants with the fastest height growth of culms in the plant kingdom (Peng et al., 2013; Chen et al., 2021). It takes only 45 to 60 days for a bamboo culm to reach a height of 15–20 meters from its initial emergence as a young shoot. Due to its wide range of uses, unique strength, and strong adaptability, Moso bamboo has become the most important bamboo resource in China. Its distribution area reaches 4.73 million hm², accounting for 70% of the total bamboo distribution area in the country (Li et al., 2024). Shengyin bamboo *(P. edulis* f. *Tubaeformis*) is a dwarf variant of Moso bamboo. Its bamboo culms have shortened internodes, with the lower part being thick and gradually expanding toward the base to form a trumpet shape, giving it a unique appearance (Wang et al., 2018; Qiu et al., 2024). Temples often use it to make trumpets; the sound produced is ethereal and celestial. It is a valuable ornamental bamboo species.

A number of studies have shown that the growth of bamboo shoots mainly depends on internode cell elongation and division (Cui et al., 2012; Tao et al., 2020; Zhang et al., 2021; He et al., 2013). Within the internode, tissues at different positions exhibit distinct growth states: tissues at the base are dominated by cell division, those in the middle by cell elongation, while those at the top cease growth (Wei et al., 2019). During the growth of Moso bamboo seedlings, exogenous application of the brassinosteroid biosynthesis inhibitor PPZ (propiconazole) or the gibberellin biosynthesis inhibitor paclobutrazol (PAC) can significantly reduce the overall plant height and internode length. In contrast, exogenous application of gibberellin (GA_3_) or auxin (NAA) promotes internode elongation of Moso bamboo seedlings (Wang et al., 2017; Zhang et al., 2018, 2020). A comparison between *Pseudosasa japonica* and its slow-growing mutant with abnormal internode development, *Pseudosasa japonica* (Sieb. et Zucc.) Makino var. *tsutsumiana* Yanagita (sgv), revealed that the brassinosteroid biosynthesis deficiency in the mutant results in the low-abundance expression of genes related to cell wall modification, biosynthesis, and cell division, ultimately leading to abnormal internode growth and development (Gao et al., 2021). Previous comparative transcriptomic studies revealed that the abnormal expression of genes related to cell wall relaxation-related enzymes, the cellulose and lignin biosynthesis pathways, as well as genes involved in hormone biosynthesis and signal transduction in Shengyin bamboo is a key factor contributing to its dwarfism. However, the epigenetic mechanisms regulating the expression of these genes remain unknown, and the screened key functional genes have not yet undergone functional validation (Wang et al., 2018).

Currently, there are existing studies on DNA methylation in aspects such as abiotic stress response, shoot growth and development, rhizome development, and flowering in bamboo (Ding et al., 2024, 2022; Yu et al., 2025). Analysis of the genomic DNA methylation profiles of *Bonia amplexicaulis* during stem growth stages (ST1-ST5) revealed that the CG and CHG methylation levels during the rapid stem growth stages (ST2-ST4) were significantly lower than those in the incubation (ST1) and plateau stages (ST5). In contrast, CHH methylation was not involved in rapid stem growth; instead, it gradually accumulated in transposable element (TE) regions and was closely associated with stem developmental time (Niu et al., 2022). Methylation analysis of single internodes (basal and middle parts) of Moso bamboo shoots at different heights revealed that 2-meter Moso bamboo shoots have a higher proportion of N6-methyladenine (m6A) modification at the RNA methylation level, while 4-meter Moso bamboo shoots have a higher 5-methylcytosine (5mC) level at the DNA methylation level (Li et al., 2023). When treated with the RNA methylation inhibitor (DZnepA) and DNA methylation inhibitor (5-azaC), the root length of Moso bamboo seedlings becomes shorter, but the number of lateral roots increases (Liufu et al., 2023). However, in Shengyin bamboo, whether the internode shortening is caused by DNA methylation regulation remains unclear.

This study first conducted a comparison of internodes between Shengyin bamboo and Moso bamboo at the anatomical level, and found that the internode shortening of Shengyin bamboo is caused by abnormal cell division and elongation. Subsequently, a large number of differentially expressed genes (DEGs) in Shengyin bamboo were identified at the transcriptomic level, including those related to DNA methylation, cell cycle, and cell wall synthesis, whose abnormal expression may contribute to the internode shortening of Shengyin bamboo. Next, we compared the two bamboo species at the DNA methylation level and demonstrated that DNA methylation plays an important role in regulating gene expression. Finally, we performed functional verification of *GRF10* (Growth-regulating factor 10), a gene highly expressed in Moso bamboo under DNA methylation regulation. This study provides new insights and evidence for understanding the epigenetic regulatory mechanisms underlying the dwarfing of Shengyin bamboo.

## Materials and methods

### Sample collection

Bamboo shoots of Shengyin bamboo and Moso bamboo were collected from the China Bamboo Expo Garden in Anji County, Zhejiang Province. The planting and management conditions were consistent between the two bamboo species. Both stands were located on flat terrain with similar forest densities, and there were no surrounding trees causing shading or growth inhibition. We selected the 15th internode from the basal part as the experimental material. Specifically, for Moso bamboo, samples included the base of the internode at the early developmental stage (M1, representing the cell division stage, 4 cm in length) and the base of the internode at the late developmental stage (M2, representing the cell elongation stage, 15 cm in length). For Shengyin bamboo, two corresponding internode samples, S1 and S2, were collected. The harvested internode samples were divided into two parts: those intended for paraffin sectioning were preserved in Formaldehyde-acetic acid-alcohol buffer (FAA), while those for transcriptome and methylation sequencing were flash-frozen in liquid nitrogen and stored at -80 °C. The formula for the FAA solution is 90 mL of 70% ethanol + 5 mL of formaldehyde + 5 mL of glacial acetic acid.

### Observation by scanning electron microscopy

The mature culms of Moso bamboo and Shengyin bamboo were observed using SEM. Following fixation in 2.5% glutaraldehyde, the tissues were rinsed four times with 0.1 M PBS. Samples were then dehydrated through a graded ethanol series and substituted with isoamyl acetate (four times). Subsequently, all bamboo samples were dried using a CO_2_ critical point dryer (Quorum EMS850, London, UK) and sputter-coated with gold (EIKO IB-3, Japan). Images were captured with an EDAX JSM-6360LV scanning electron microscope (Japan) at an accelerating voltage of 2 kV.

### Paraffin section preparation and observation

Bamboo shoot tissues were fixed in FAA for more than 48 hours, followed by serial ethanol dehydration with concentrations of 30%, 50%, 70%, 90%, 95%, and 100%, and xylene clearing, before being embedded in paraffin (Li et al., 2025). Sections were cut using a Leica RM 2235 rotary microtome with a thickness of 7-8 μm, yielding both longitudinal and cross-sections of the bamboo shoots. The sections were stained by the safranin-fast green staining method, mounted with neutral balsam, and finally observed and photographed using a Leica DM2500 optical microscope.

### Transcriptome sequencing

Total RNA was extracted from the two bamboo species using the RNAprep pure Plant Kit (RNAprep pure Plant Kit; cat. no. DP441; Tiangen), and RNA was treated with DNase I to remove any residual genomic DNA. The quality of the total RNA was then checked by electrophoresis on a 1% (w/v) agarose gel. A Nanodrop 2000 Spectrophotometer (Thermo Scientiffc) was used to check RNA concentration and purity, while an Agilent 2200 System (Agilent 2200 Bioanalyzer) was used to determine RNA integrity. Total RNA that passed all quality checks was used for library construction. Strand-speciffc RNA-seq libraries were constructed using the dUTP method and sequenced on an Illumina Novaseq 6000 instrument. The raw sequencing reads were processed according to the following procedures. First, fastp (Chen et al., 2018) was employed for quality control (QC) and to remove adapter sequences. Next, FASTQ ffles containing the quality-controlled reads were aligned to the Moso bamboo genome (Zhao et al., 2018) using Hisat2 (Kim et al., 2019) with default options. Only reads that aligned to a single region in the genome were used for downstream analysis. Gene expression quantification was performed using FPKM (Fragments Per Kilobase Million). Differential gene expression between sample groups was analyzed with the DESeq2 method (≥1-fold change, FDR <0.01) to identify sets of differentially expressed genes (Love et al., 2014). Differentially expressed genes were annotated and classified via BLASTX searches against the Swiss-Prot (Boeckmann et al., 2003), Pfam protein domain (Finn et al., 2008), Gene Ontology (Ashburner et al., 2000), and KEGG (Kanehisa et al., 2025) databases.

### Whole genome bisulfite sequencing

Total genomic DNA (gDNA) was extracted from the two bamboo species using the DNeasy Plant Mini Kit (QIAGEN 69104, QIAGEN, Germany). The quality of the genomic DNA was assessed by agarose gel electrophoresis and UV spectrophotometry. The gDNA was sheared into fragments of 100–500 bp using a BioRuptor (Diagenode, Belgium). Following end repair, adenylation, and adapter ligation (to protect against bisulfite-induced degradation), the DNA fragments were treated with bisulfite using the Zymo Methylation Kit (Zymo Research, USA). The treated DNA was purified on spin columns and used for sequencing library preparation. During this process, bisulfite-treated single-stranded DNA was subjected to random priming using a polymerase capable of reading uracil nucleotides to synthesize DNA containing specific sequence tags. These tags were then used to add adapter sequences to the 5’ and 3’ ends of the original DNA fragments via PCR. The epigenomic libraries were diluted and loaded onto a cBot DNA cluster generation system. After cluster generation was completed, the two samples were transferred to an Illumina Novaseq 6000 platform for sequencing. All steps were performed according to the manufacturers’ instructions.

Raw sequencing data were first processed to remove reads containing adapter sequences, unknown bases, or low-quality bases. The resulting clean reads were uniquely mapped to the Moso bamboo reference genome using the alignment software Bowtie2 (Langmead and Salzberg, 2012). The BS-Seq data were used to detect the methylation status of individual cytosines, the number of methylated cytosine sites, and the methylation proportion per genomic context. The methylation level was calculated as the number of reads for each methylated cytosine (mC) divided by the total reads for that cytosine. Differentially methylated regions (DMRs) were defined as regions containing at least 5 CG (CHH or CHG) sites with a 2-fold change in methylation level (Fisher’s exact test, P ≤ 0.05) (Hebestreit et al., 2013). Basic data manipulation and statistical analysis were performed using R packages. All the sequencing data were uploaded to NCBI under accession numbers PRJNA1371697.

### qRT-PCR analysis

Total RNA was extracted from frozen leaves using the RNA Easy Fast Kit (DP452, TIANGEN, Beijing, China) according to the manufacturer’s protocol. First-strand cDNA synthesis was performed with the PrimeScript™ RT Reagent Kit (TaKaRa, Kyoto, Japan). Quantitative real-time PCR (qRT-PCR) reactions were carried out using the SYBR™ Green Premix Pro Taq HS qPCR Kit (Accurate Biology, Hunan, China) on an ABI StepOne System (Thermo Fisher Scientific, Waltham, MA, USA). Gene-specific primers were designed with Oligo 7 software (**Supplementary Table S1**). Tonoplast intrinsic protein 41 (*TIP41*) was employed as the internal reference gene (Zhou et al., 2023). The relative expression levels of each gene were calculated using the 2^−ΔΔCt^ method.

### Overexpression of *PheGRF10* (Ped09CXg23240)

The full open reading frame of the PheGRF10 was amplified from cDNA using the primer pairs as follows: 5′-ATGGACCTGG GCGGGATGG-3′ (forward) and 5′-TCACACCAGGCGGATGCT CG-3′ (reverse). The amplified product was then cloned into the pMD18-T Vector. Subsequently, the PheGRF10 coding sequence was inserted into the Cambia1301::PhePheGRF10 vectors, driven by the CaMV 35S promoter using the homologous recombination method. The recombinant vector was then introduced into rice (Oryza sativa L. subsp. japonica cv. Zhonghua 11) via Agrobacterium tumefaciens (EHA105)-mediated transformation following the method of Hiei et al. (1994). After extensive phenotypic characterization, PCR confirmations, and kanamycin selection, fifteen independent T3-generation transgenic lines were successfully established. The first, second, third, and fourth internodes from the base to the top of the stem in both transgenic lines and wild-type plants were collected as samples for paraffin sectioning. The middle portion of each internode was selected for section preparation, following the same methods described in Section 2.1.

## Results

### Comparison of anatomical structures

**Figure 1:**
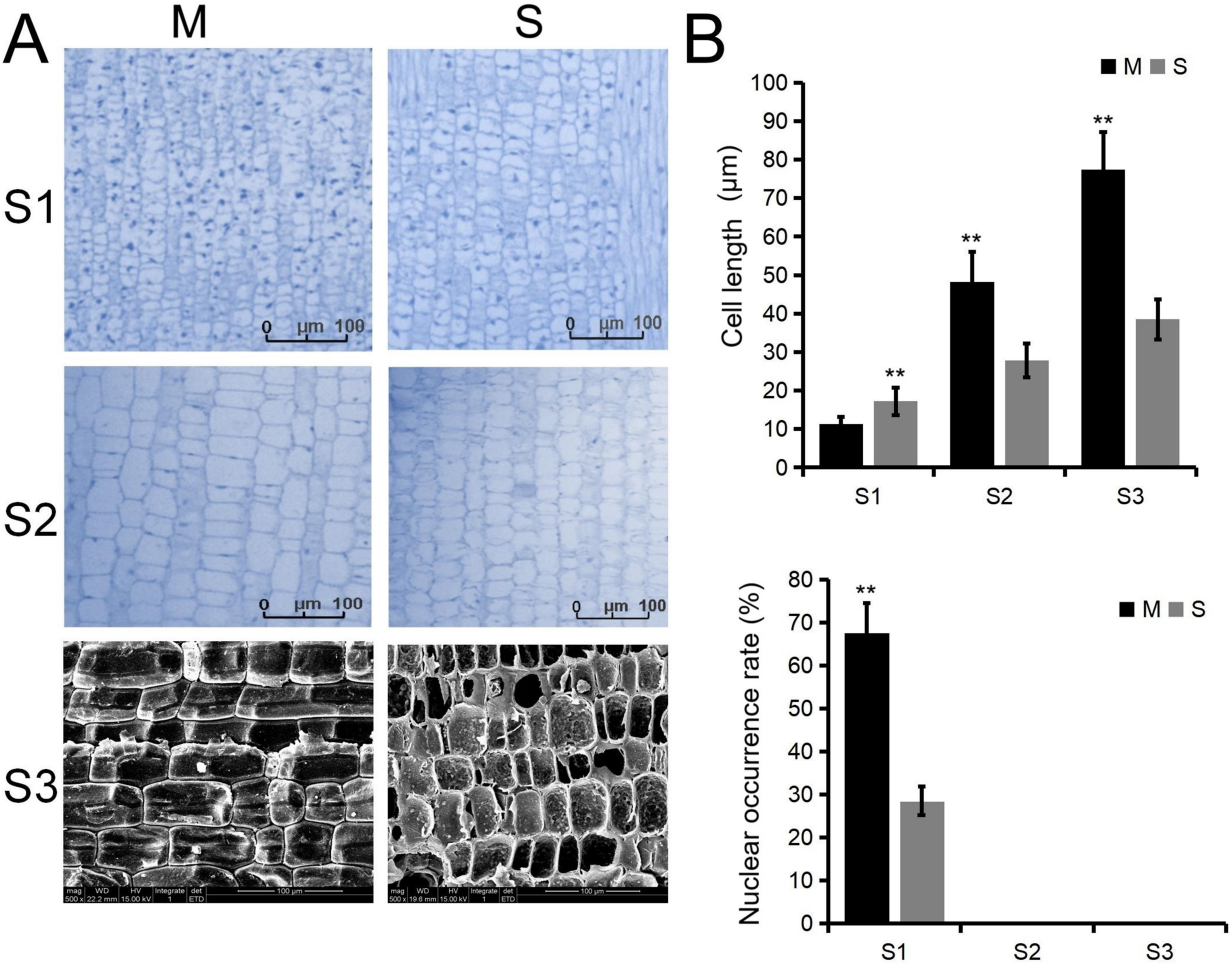
Observation of anatomical structure and statistics of cell length and nuclear occurrence rate in the 15th internode of Moso bamboo (M) and Shengyin bamboo (S). **(A)** Observation of longitudinal anatomical structure of internodes, **(B)** statistics of cell length (Upper panel) and statistics of nuclear occurrence rate (Lower panel).

We first compared the internode lengths of Moso bamboo and Shengyin bamboo. In the early stages of internode development, the nuclei occurrence rate at the base of Moso bamboo internodes was significantly higher than that of Shengyin bamboo (**Figure 1A**). In the later stages of development, Moso bamboo entered the cell elongation phase, and its cell length became significantly longer than that of Shengyin bamboo (**Figure 1B**). Upon stem maturation, the internode cell length of Moso bamboo was more than twice that of Shengyin bamboo. This indicates that the shortened internodes in Shengyin bamboo are due to the combined effects of abnormal cell division and elongation.

### Transcriptome sequencing identifies key genes with abnormal expression that cause the dwarfing of Shengyin bamboo

**Figure 2:**
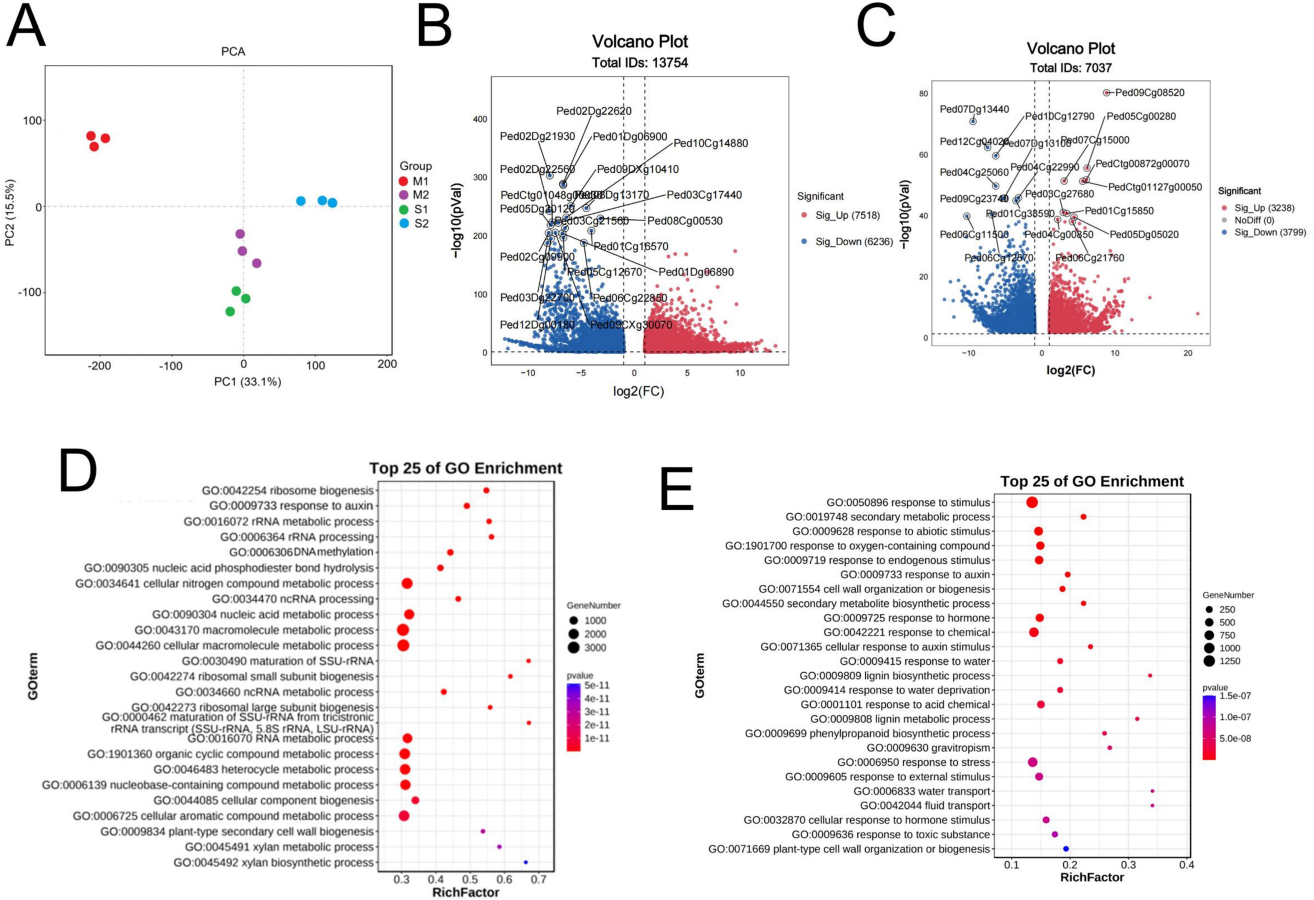
Transcriptome data analysis. **(A)** Principal Component Analysis (PCA) among various samples; **(B)** Analysis of Differentially expressed genes (DEGs) between M1 and S1; **(C)** Analysis of DEGs between M2 and S2; **(D)** GO functional annotation of DEGs between M1 and S1; **(E)** GO functional annotation of DEGs between M2 and S2.

To identify key genes underlying the dwarfism of Shengyin bamboo, we conducted a comparative transcriptome analysis of Moso bamboo and Shengyin bamboo. The results of Principal Component Analysis (PCA) based on gene expression levels showed that M1 (representing the early internode development stage of Moso bamboo) exhibited significant differences from M2 (the late internode development stage of Moso bamboo) and the two internode samples of Shengyin bamboo (S1 and S2) (**Figure 2A**; **Supplementary Table S2**). Statistics on the number of differentially expressed genes (DEGs) revealed that a total of 13,754 genes were differentially expressed between M1 and S1, among which 7,518 were highly expressed in moso bamboo and 6,236 were highly expressed in Shengyin bamboo (**Figure 2B**, **Supplementary Table S3**). At the late stage of internode development, a total of 7,037 genes were differentially expressed between M2 and S2, including 3,238 genes highly expressed in Moso bamboo and 3,799 genes highly expressed in Shengyin bamboo (**Figure 2C**, **Supplementary Table S4**). Functional annotation (GO) of DEGs indicated that genes related to ribosome biogenesis, response to auxin, rRNA metabolic process, and DNA methylation were differentially expressed between M1 and S1 (**Figure 2D**), while genes involved in response to stimulus, secondary metabolic process, and response to abiotic stimulus were differentially expressed between M2 and S2 (**Figure 2E**). In addition, genes associated with response to auxin, cell wall organization or biogenesis, and response to hormone also showed differential expression. GO annotation results indicated that the cell division-related genes (e.g., those associated with ribosome biogenesis) and DNA methylation-related genes exert a key function in the early stage of Moso bamboo shoot growth, while genes related to endogenous hormones and cell wall synthesis play important roles in the late stage.

### WGBS analysis

**Figure 3:**
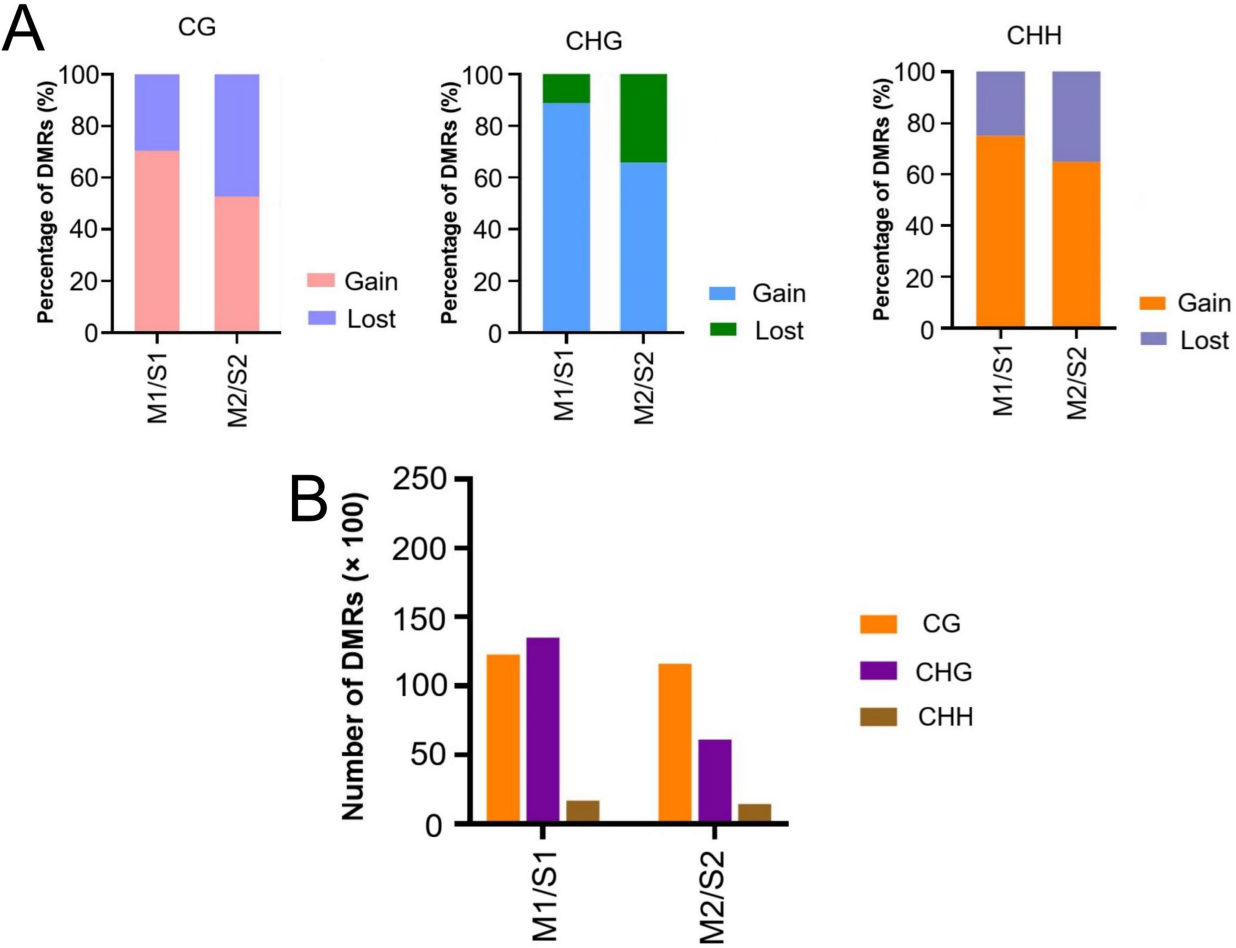
Quantitative and classification analysis of differential DNA methylated regions. **(A)** Percentage of gain or loss of differentially methylated regions (DMRs) based on paired comparisons; **(B)** Number of CG, CHG, and CHH DMRs in different paired comparisons of the four samples.

Transcriptomic results demonstrate that a large number of DNA methylation-related functional genes are differentially expressed between Moso bamboo and Shengyin bamboo, suggesting that the differences in DNA methylation levels of bamboo shoots between Shengyin bamboo and Moso bamboo may be a key factor contributing to the dwarfism of Shengyin bamboo. Therefore, we compared the global DNA methylation levels between Moso bamboo and Shengyin bamboo. DMRs from pairwise comparisons (M1 versus S1, M2 versus S2) were dominated by methylation gains in Moso bamboo (**Figure 3A**). However, the methylation gains of moso bamboo in the early stage were significantly higher than those in the late stage. Statistics on the number of DMRs revealed that among M1 and S1, the most prevalent type of differential methylation was CHG, followed by CG. In contrast, the opposite pattern was observed between M2 and S2: the most common type of differential methylation was CG, followed by CHG (**Figure 3B**).

**Figure 4:**
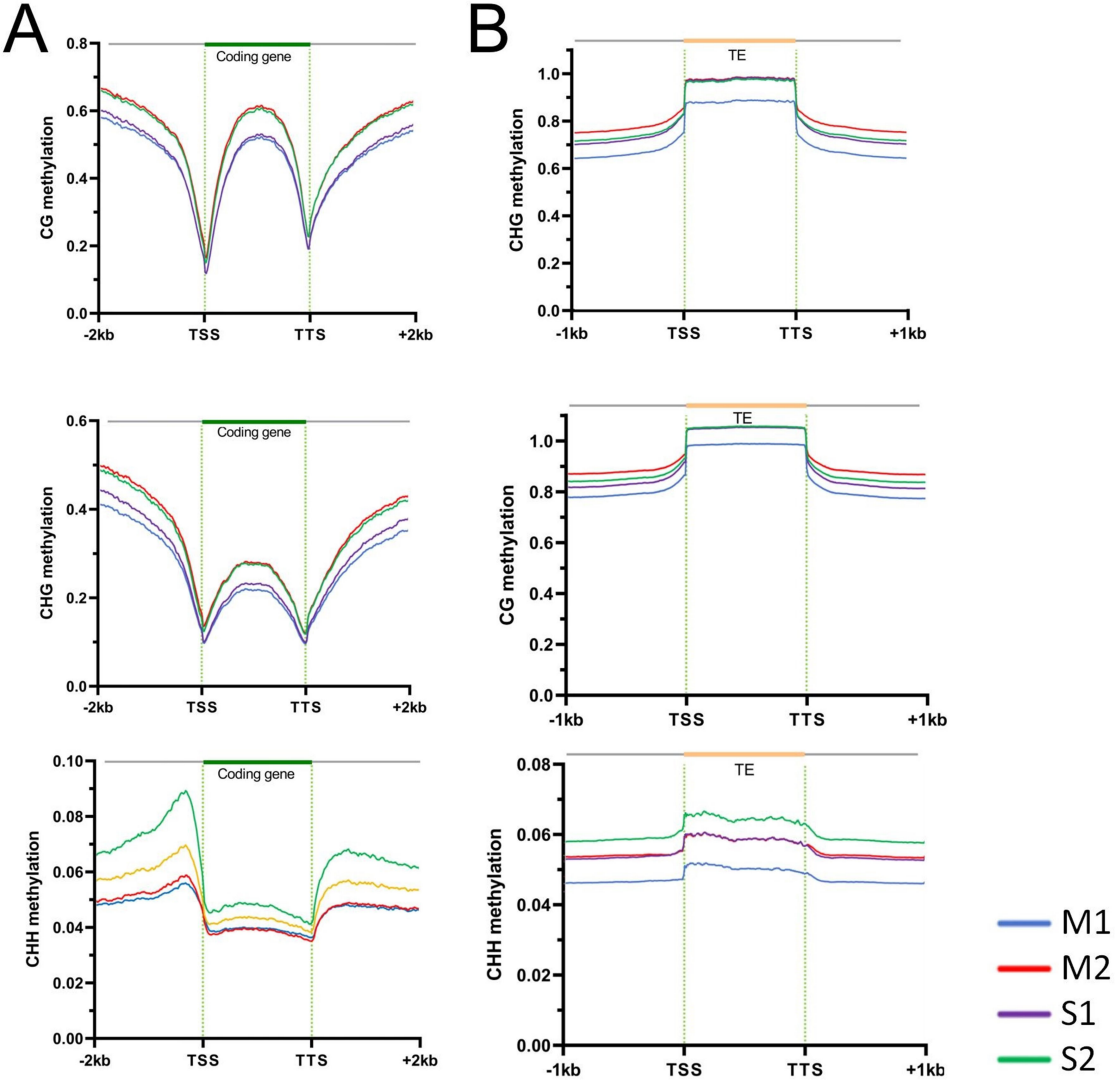
Different types of DNA methylation levels of protein-coding genes **(A)** and transposons (TEs) **(B)**. TSS, transcription start site; TTS, transcription termination site.

CG methylation followed a bell-shaped distribution over protein-coding genes and their ±2 kb flanking regions (**Figure 4A**). CG methylation levels were the lowest at the transcription start sites (TSSs) and transcription termination sites (TTSs), with a slightly higher level around TTSs than TSSs. The profile for CHG methylation was similar to that for CG methylation, but with smaller fluctuations within the transcribed regions. By contrast, the distribution of CHH methylation was almost uniform across the transcribed regions. However, the upstream promoter regions of protein coding genes had higher CHH methylation levels than the downstream regions (**Figure 4A**). Besides, we detected high levels of DNA methylation within the body of TEs (**Figure 4B**), while the methylation levels of the flanking regions were lower. In addition, the DNA methylation levels in the gene body, TE body, and their upstream and downstream regions were higher at the late stage of shoot growth (M2 and S2) than those at the early stage (M1 and S1).

**Figure 5:**
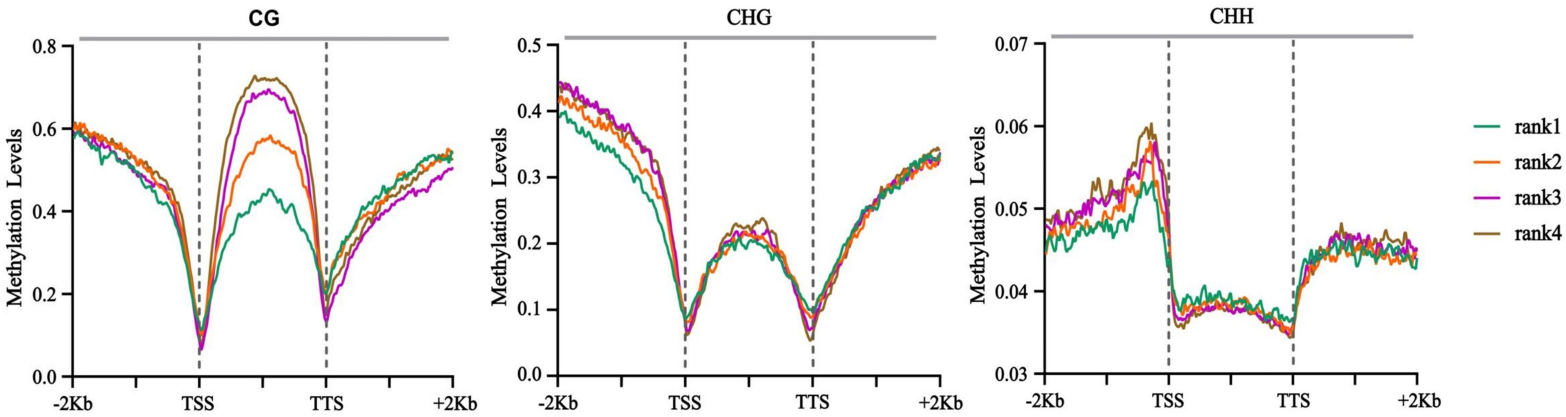
Characteristics of DNA methylation levels associated with differences in gene expression levels. The x-axis represents the position and level of methylation sites within the gene body and its upstream and downstream 2kb regions; the y-axis indicates the methylation level. Genes are divided into rank4, rank3, rank2, and rank1 based on their expression levels from low to high.

**Figure 6:**
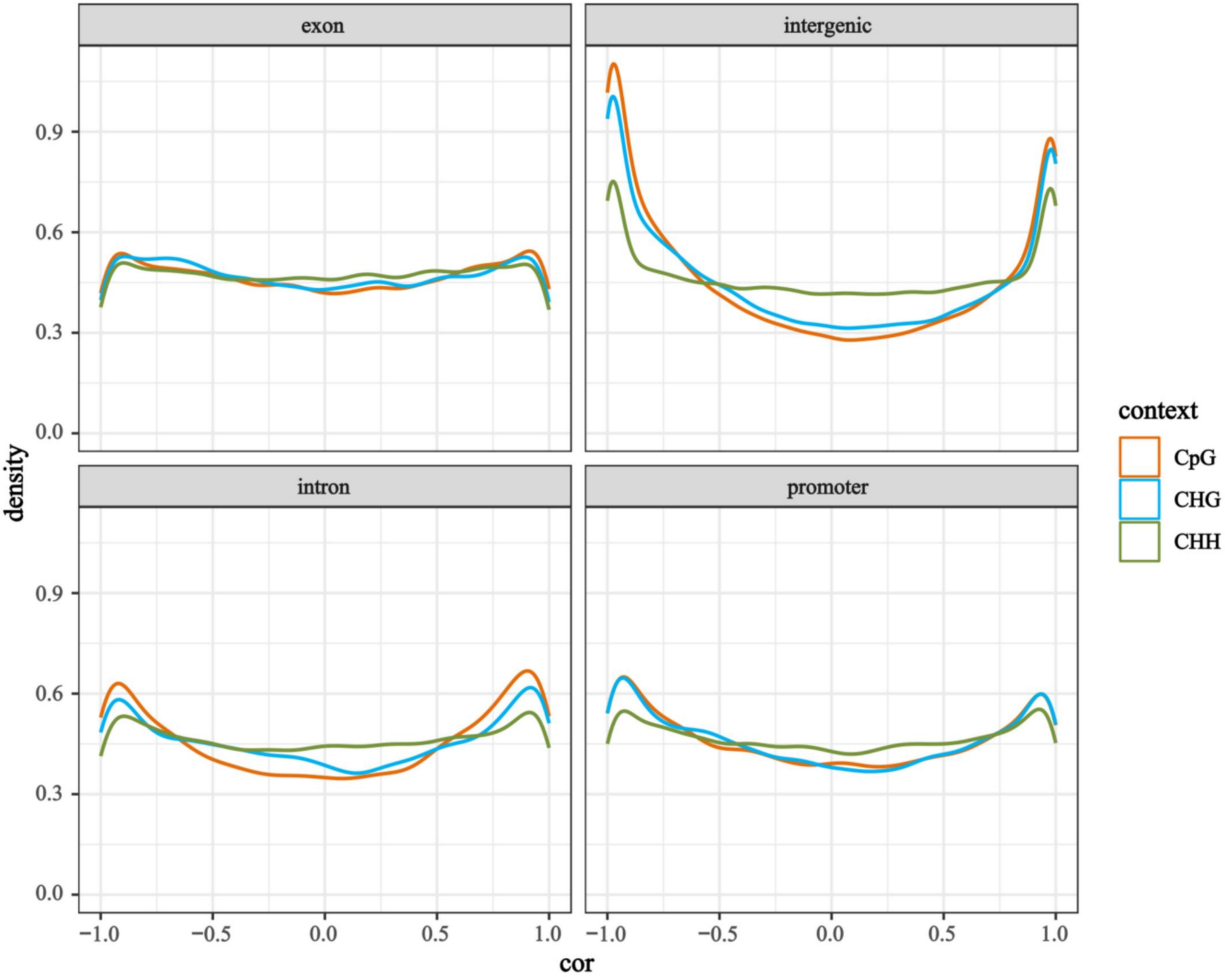
Density distribution plot of correlation coefficients between DNA methylation types (CpG, CHG, CHH) and gene expression across different genomic regions. The x-axis represents the correlation coefficient between methylation level and gene expression, while the y-axis indicates the density (reflecting the frequency of occurrence of a particular correlation coefficient).

We divided the gene expression levels into four categories (Flank1-4) from low to high. Overall, the methylation levels of gene promoters and gene bodies showed a negative correlation with gene expression, i.e., the higher the gene expression level, the lower the DNA methylation level (**Figure 5**). Among these, the levels of CHG and CHH methylation in promoters have a greater impact on gene expression compared with that of CG methylation, whereas the level of CG methylation in gene coding regions exerts a more significant influence on gene expression. Correlation analysis between DNA methylation types (CG, CHG, CHH) and gene expression in different genomic regions revealed that the methylation levels in the intergenic regions, promoters, and intronic regions of most genes showed significant positive or negative correlations with gene expression, whereas the methylation levels in exonic regions had no obvious correlation with gene expression (**Figure 6**). These results indicate that the methylation levels of genes and their surrounding regions significantly promote or inhibit gene expression.

**Figure 7:**
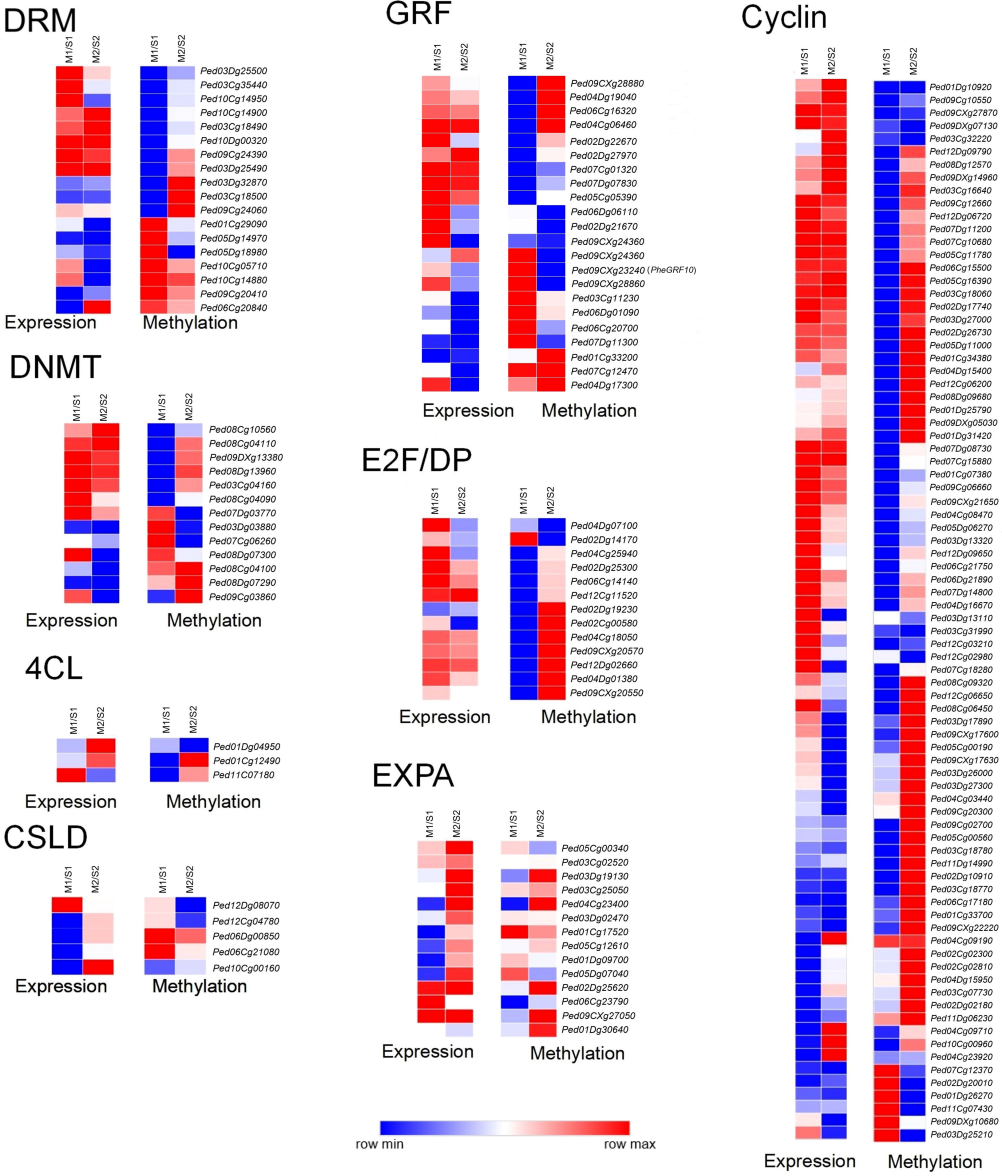
Analysis of differential expression and DNA methylation levels of *DRM*, *DNMT*, *GRF*, *E2F/DP*, *EXPA*, *Cyclin*, *4CL*, *CSLD* family genes.

**Figure 8:**
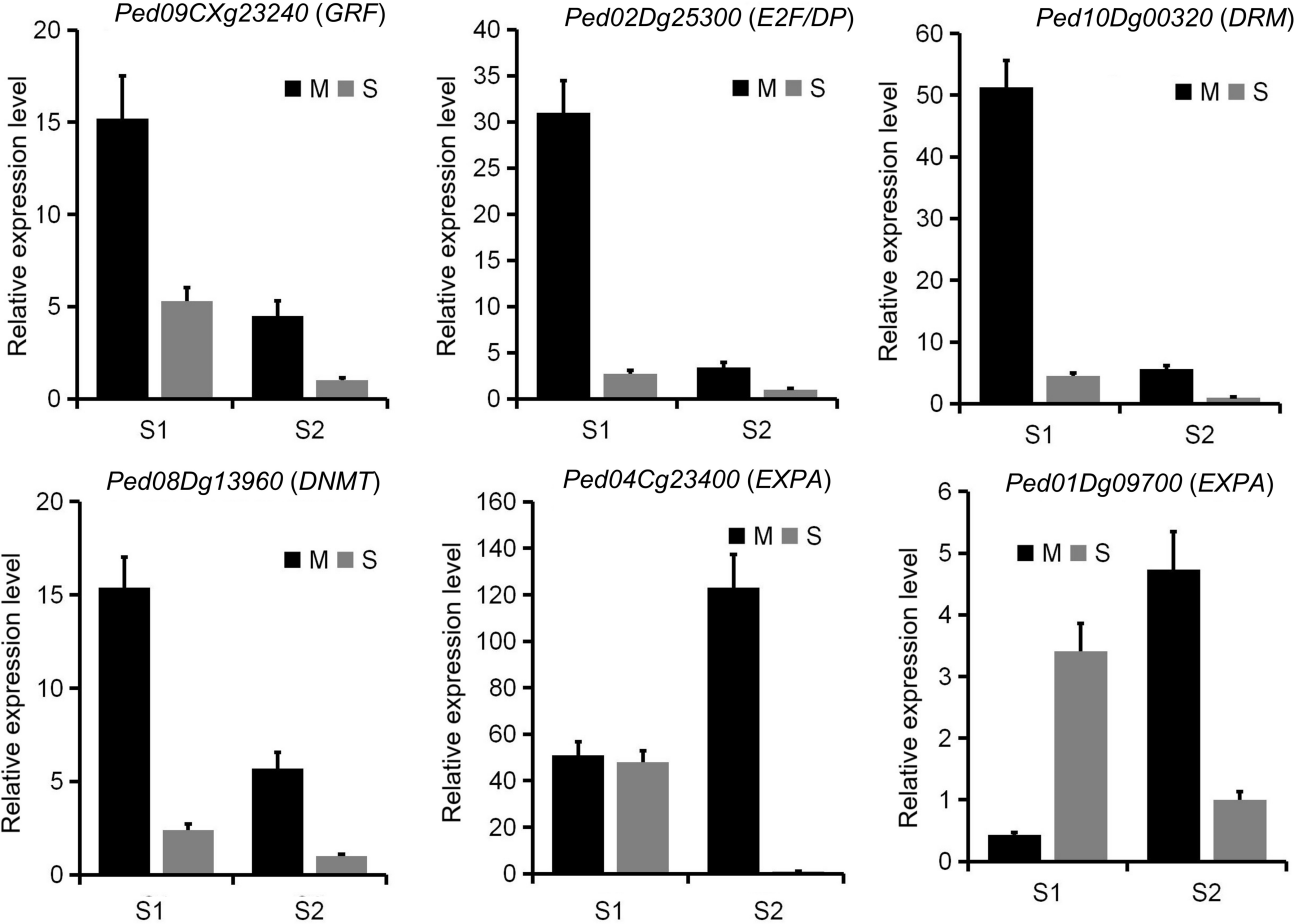
Expression analysis of key genes regulating internode elongation in Moso bamboo and Shengyin bamboo via qRT-PCR. M and S represented for Moso bamboo and Shengyin bamboo, respectively.

Based on the functional annotation of differentially expressed genes, we selected several key gene families for further gene expression and promoter methylation levels analysis (**Figure 7**; **Supplementary Table S5**). These families encompass those involved in DNA methylation regulation (Domains Rearranged Methyltransferase, *DRM*; DNA Methyltransferase, *DNMT*), cell cycle control (Cyclin; *E2F* transcription factor/Dimerization Partner, *E2F/DP*), cell wall synthesis and growth (Expansin-like A, *EXPA*; Cellulose Synthase-Like D, *CSLD*; 4-Coumarate: CoA Ligase, *4CL*), and general cell division and growth (*GRF*). Most genes in the *DRM*, *DNMT*, *cyclin* and *GRF* families showed significantly higher expression levels in Moso bamboo than in Shengyin bamboo during the early stages of internode growth, while no significant difference was observed in the later stages. The promoter methylation levels of most genes in these families exhibited a negative correlation with their corresponding gene expression. Among the *GRF* family, *Ped09CXg23240* (*PheGRF10*) exhibited significantly higher expression levels in Moso bamboo than in Shengyin bamboo during both developmental stages, with significant differences observed (**Figure 8**). Furthermore, this expression pattern was negatively correlated with its promoter methylation levels. *E2F/DP* exhibits higher expression patterns in Moso bamboo both at the early and late stages of internode development, with the difference in expression between Moso bamboo and Shengyin bamboo being more significant in the early stage. Analysis of promoter methylation levels showed that the DNA methylation levels of the vast majority of genes were higher in Shengyin bamboo at the early stage, showing a negative correlation with gene expression. In contrast, there was no significant correlation between gene promoter methylation levels and gene expression at the late stage. Most members of the *EXPA* family are highly expressed in Moso bamboo at the late stage of internode development. The vast majority of members show a significant negative correlation between the DNA methylation level of the promoter and gene expression at the early stage of internode development, while there is no significant correlation between methylation and gene expression at the late stage. For instance, both *Ped01Dg09700* and *Ped04Cg23400* exhibited higher expression in Moso bamboo than in Shengyin bamboo at the late stage of internode elongation (**Figure 8**). However, the expression of *Ped01Dg09700* was positively correlated with its promoter methylation level, while that of *Ped04Cg23400* showed a negative correlation with its promoter methylation level. In addition, we also analyzed two cellulose synthesis-related gene families (*CSLD* and *XTH*). Genes of these two families exhibit higher expression in Moso bamboo than in Shengyin bamboo at the late stage of internode development, and the gene expression of more than half of the *CSLD* and *XTH* members shows a negative correlation with the DNA methylation levels of their promoters.

### Functional verification of *PheGRF10* (*Ped09CXg23240*)

**Figure 9:**
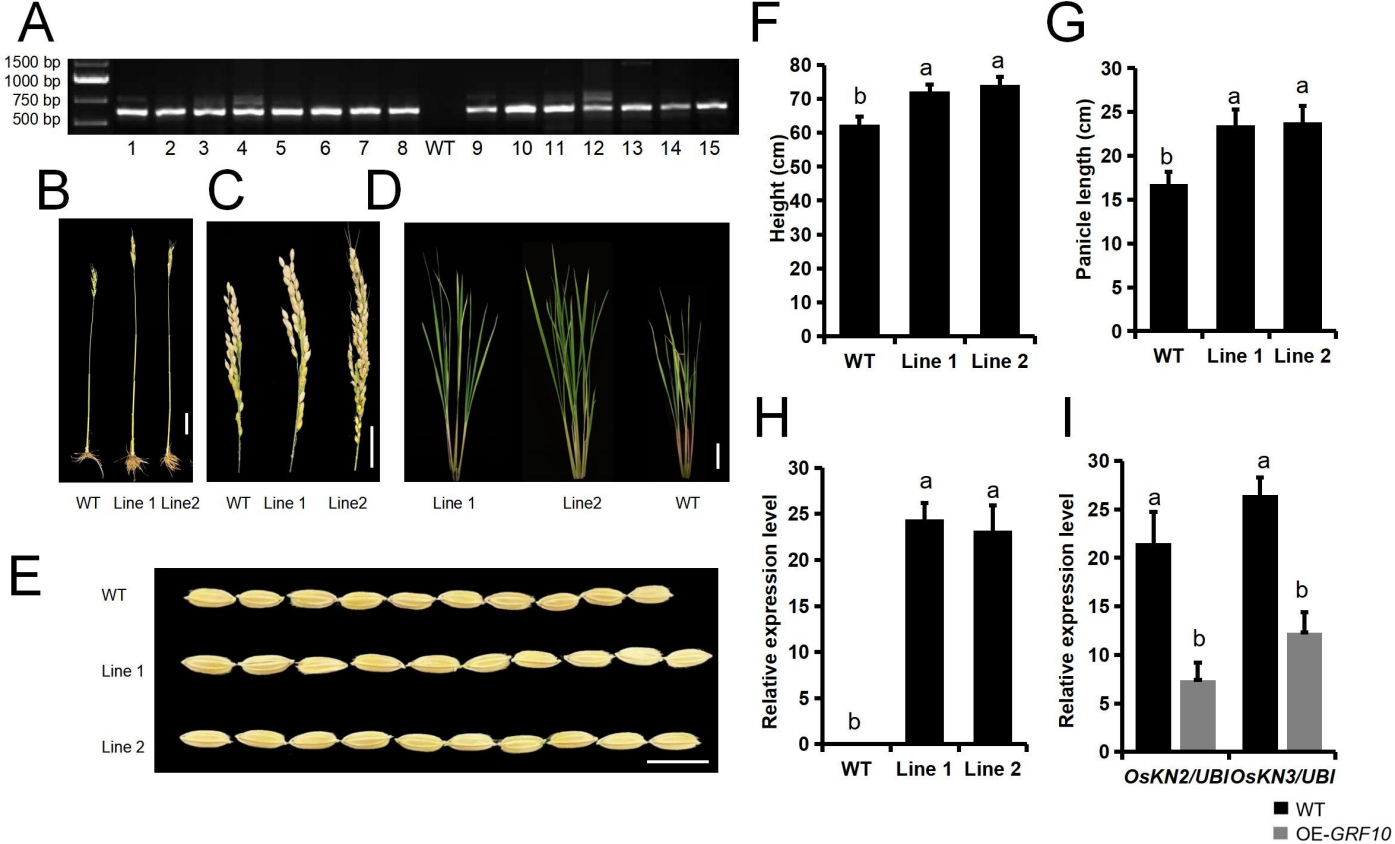
Phenotypic observation of *PheGRF10*-overexpressing rice lines. **(A)** Semi-quantitative PCR detection of the *PheGRF10*-overexpressing rice lines. **(B)** Observation of main stem phenotype in transgenic lines, scale bar, 10 cm. **(C)** Observation of panicle phenotype in transgenic lines, scale bar, 5 cm. **(D)** Observation of leaf phenotype in transgenic lines, scale bar, 10 cm, **(E)** Observation of seed length, 1 cm. **(F)** Statistics of plant height in overexpressing lines. **(G)** Statistics of panicle length in transgenic lines. **(H)** Expression level of *PheGRF10* in rice overexpression lines. **(I)** qRT-PCR analysis of downstream KNOX gene expression.

**Figure 10:**
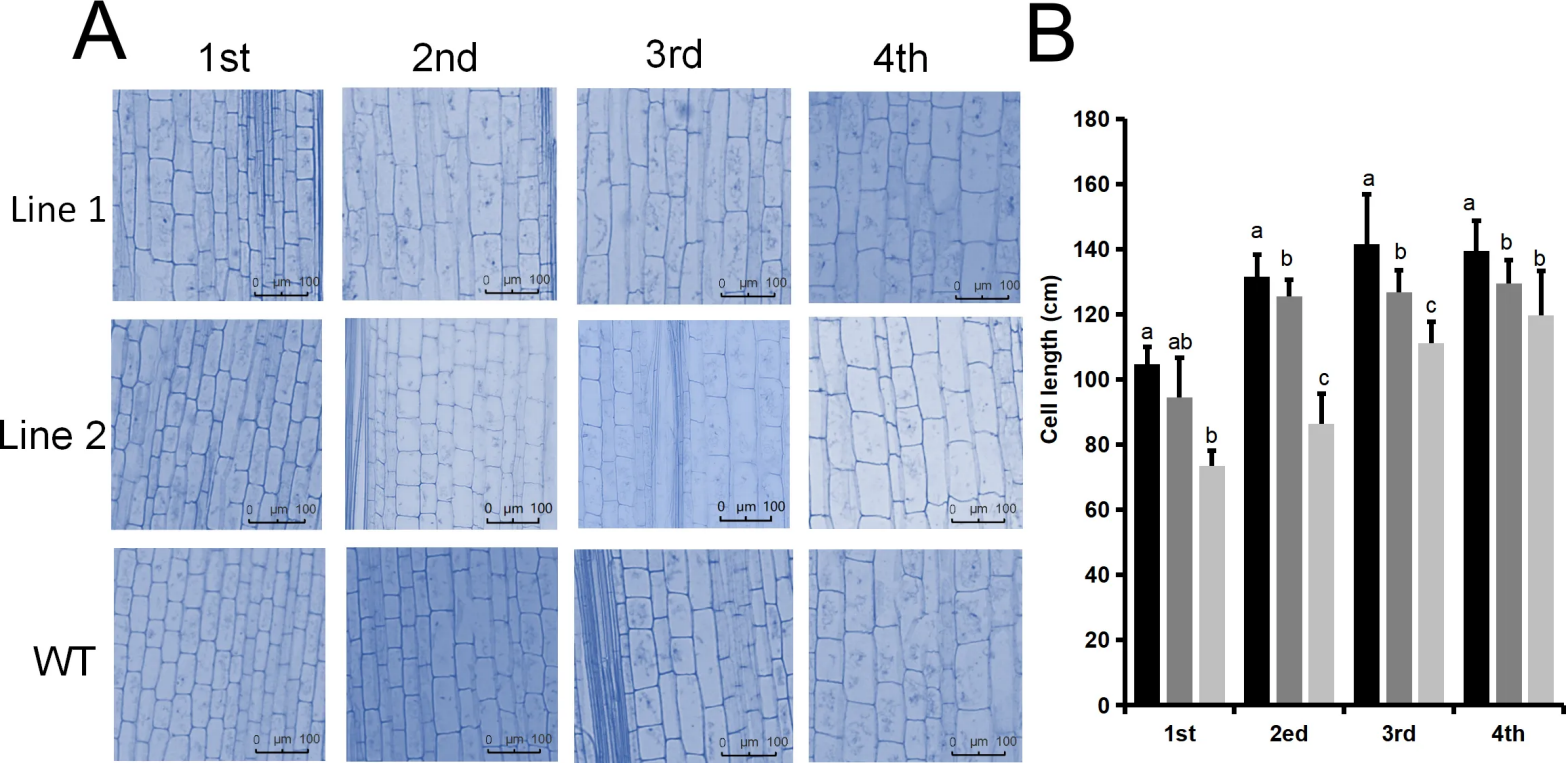
Observation of stem anatomical structures in *PheGRF10*-overexpressing rice lines. **(A)** Observation of stem anatomical structure and **(B)** Statistics of cell length. 1st, 2nd, 3rd, and 4th represent the 1st, 2nd, 3rd, and 4th internodes from the base upwards of rice stems.

Based on transcriptome data, we identified the key gene *PheGRF6a* (*Ped09CXg23240*) that is potentially involved in internode cell elongation of Moso bamboo. Following hygromycin selection and semi-quantitative PCR analysis, a total of 15 transgenic rice lines were obtained (**Figures 9A**). Overexpression of *PheGRF10* in rice cultivar ‘Zhonghua 11’ revealed that, compared with the wild type (WT), the plant height of the main stem in overexpression lines was significantly higher, with significant statistical differences (**Figures 9B, F**). In addition, the panicles, seeds, and leaves of the overexpression lines were significantly longer than those of the WT (**Figures 9B–G**). qRT-PCR results showed that *PheGRF10* was highly expressed in transgenic rice, while no expression was detected in the WT (**Figures 9H**). Furthermore, the high expression of *PheGRF10* inhibited the expression levels of rice *KNOX* family genes (*KN1* and *KN2*) in transgenic rice (**Figures 9I**). Observation of the anatomical structure of transgenic stems showed that the lengths of internode cells of the over-expression lines were longer than those of the WT (**Figures 10A, B**).

## Discussion

DNA methylation is a key epigenetic modification that regulates plant growth and development by modulating gene expression (Xi et al., 2023; Jiang et al., 2025; Madzima et al., 2021; He et al., 2022). WGBS revealed distinct DNA methylation patterns between Moso bamboo and Shengyin bamboo, providing direct evidence for epigenetic regulation of bamboo dwarfism. The distribution patterns of CG, CHG, and CHH methylation in protein-coding genes and transposons (TEs) observed in this study are consistent with the conserved methylation profiles in angiosperms (Zhou et al., 2024; Zhang et al., 2020). CG and CHG methylation showed bell-shaped distributions across gene bodies, with minima at transcription start sites (TSSs) and transcription termination sites (TTSs)—a pattern associated with transcriptional regulation. CHH methylation, by contrast, was enriched in promoter regions, which may be related to the repression of transposon activity and maintenance of genome stability (Du et al., 2024). At the late developmental stage of Moso bamboo, the DNA methylation levels in gene bodies, transposon (TE) bodies, and their flanking regions were higher. However, the differences between the early and late stages in Shengyin bamboo were not as significant as those in Moso bamboo. This indicates that DNA methylation is dynamically regulated during internode development and may be involved in the transition from cell division to cell elongation.

Changes in DNA methylation patterns can lead to alterations in chromatin structure and gene accessibility, ultimately influencing gene expression (Zhang et al., 2015; Wang et al., 2021). The CG methylation levels of genes are positively correlated with their expression levels, while many highly expressed genes exhibit low CG methylation levels (Zilberman et al., 2007; Takuno and Gaut, 2012). In this study, we obtained similar results: compared with CHG and CHH, the inhibitory effect of CG methylation levels in gene bodies on gene expression is more significant. This indicates that CG levels play an important role in regulating gene expression during the internode elongation of Moso bamboo shoots.

Studies have shown that the internode elongation of bamboo shoots is the result of the combined effect of cell division and cell elongation (Li et al., 2018; Wei et al., 2019; Yang et al., 2022). We compared the early and late stages of internode development between Moso bamboo and Shengyin bamboo separately. We found that Moso bamboo exhibits more distinct stage-specific characteristics in internode development: its growth is dominated by cell division in the early stage and by cell elongation in the late stage. Gene families with different functions fulfill distinct roles in Moso bamboo during the early and late stages of internode elongation. In rice, when *OsGRF1* expression is knocked down by RNA interference (RNAi), it results in reduced leaf size, dwarf plant height, and delayed heading date (Luo et al., 2005). Overexpression of *GRF5* in diploid Populus leads to transgenic lines with larger leaves and faster growth; in addition, both stem diameter and plant height of the transgenic lines are increased (Wu et al., 2021). In the present study, most *GRF* genes were found to be highly expressed during the early stage of internode elongation in Moso bamboo, showing a negative correlation with methylation levels; furthermore, heterologous overexpression of *PheGRF10* in rice significantly promoted stem height growth, collectively indicating that these genes, under the regulation of DNA methylation, enhance internode elongation in Moso bamboo. Additionally, members of the *E2F/DP* gene family—associated with cell proliferation and the cell cycle (Li et al., 2021; Heckmann et al., 2011) —exhibit higher expression patterns in Moso bamboo than in Shengyin bamboo both at the early and late stages of internode development, with more significant differences observed in the early stage. This indicates that genes related to cell division and proliferation, such as *E2F/DP*, *GRF*, and *cyclin*, play important roles in internode cell division during the early stage of Moso bamboo development. In contrast, the abnormal expression of these genes in Shengyin bamboo results in abnormal internode cell division.

At the late stage of internode elongation, *EXPA*—a gene family associated with cell elongation—exhibits higher expression levels in Moso bamboo than in Shengyin bamboo (Li et al., 2024; Fan et al., 2025). In addition, overall, there were no significant differences in *EXPA* expression levels between the early and late stages in Shengyin bamboo, and its expression levels in the early stage were even higher than those in Moso bamboo. This is consistent with the results of anatomical observations: at the early stage of internode elongation, the internode cell length of Moso bamboo was actually shorter than that of Shengyin bamboo. Similarly, the *XTH* and *CSLD* families, which are involved in cell wall synthesis and cellulose synthesis, show the same expression pattern (Luan et al., 2011; Song et al., 2025; Zhang et al., 2024). This indicates that at the late stage of internode elongation, under the regulation of DNA methylation, genes related to cell wall synthesis and cell growth are highly expressed in Moso bamboo, thereby facilitating the transition of internode growth from being promoted by cell division (in the early stage) to being driven by cell elongation.

The cell division-related genes (*Cyclin*, *GRF*), and cell elongation-related genes (*EXPA*), their higher expression in Moso bamboo was accompanied by lower promoter methylation, while the opposite was true in Shengyin bamboo. All of this indicates that DNA methylation plays a significant role in regulating the internode elongation of bamboo shoots. In Moso bamboo, genes involved in internode cell division and those involved in cell elongation fulfill their functions during the early and late stages of internode development, respectively. However, in the case of Shengyin bamboo, gene expression appears to be more disordered. Many genes related to cell elongation and cell wall synthesis are highly expressed in the early stages. This irregular expression indirectly affects the internode elongation growth of Shengyin bamboo—initiating cell elongation growth before the completion of cell division growth.

## Conclusion

This study investigated the molecular mechanism underlying the dwarfism of Shengyin bamboo (a variant of Moso bamboo) by integrating anatomical, transcriptomic, and whole-genome bisulfite sequencing (WGBS) analyses. Anatomical observations revealed that the shortened internodes of Shengyin bamboo result from abnormal cell division and elongation compared to Moso bamboo, which exhibits distinct stage-specific growth (cell division-dominated early stage and cell elongation-dominated late stage). WGBS analysis identified divergent DNA methylation patterns between the two bamboo species: CG, CHG, and CHH methylation showed conserved distributions across gene bodies and transposons (TEs), with promoter and gene body methylation negatively correlating with gene expression. Key growth-related gene families (*GRF*, *E2F/DP*, *Cyclin*, *EXPA*, *CSLD*, *XTH*) exhibited ordered temporal expression in Moso bamboo, regulated by DNA methylation, while their expression was disordered in Shengyin bamboo (e.g., early high expression of cell elongation-related genes). Functional verification confirmed that over-expression of *PheGRF10* promotes cell elongation and plant height. Collectively, these results demonstrate that DNA methylation mediates the stage-specific expression of cell division and elongation-related genes, which is crucial for Moso bamboo’s rapid internode growth. The disrupted DNA methylation-regulated gene expression in Shengyin bamboo causes abnormal growth, providing novel insights into the epigenetic regulatory network of bamboo dwarfism.

## Data Availability

The datasets presented in this study can be found in online repositories. The names of the repository/repositories and accession number(s) can be found in the article/**Supplementary Material**.
