# Correction: A comparative study of phyllostachys edulis and its dwarf variant phyllostachys edulis ‘Tubaeformis’ at the anatomical, transcriptomic, and DNA methylation levels

**DOI:** 10.3389/fpls.2026.1840239

**Published:** 2026-05-29

**Authors:** Qiu Zhenhua, Sun Yuanyuan, Lin Shuyan, Liu Xinyao, Li Long

**Affiliations:** 1 National Key Laboratory for the Development and Utilization of Forest Food Resources, Nanjing Forestry University, Nanjing, China; 2 Bamboo Research Institute, Nanjing Forestry University, Nanjing, China; 3 College of Life Sciences, Nanjing Forestry University, Nanjing, China; 4 Laboratory of Pathogen Research, West China Hospital, Sichuan University, Chengdu, China

**Keywords:** DNA methylation, EXPA, Expansin-like A, GRF (growth-regulating factor), *GRF10* (Growth-regulating factor 10)

There was a mistake in **Figure 9**, page 12 as published.

The gene name *PheGRF6a* in **Figure 9I** is corrected to *PheGRF10*. During the initial submission, the gene with the locus ID Ped09CXg23240 from the third-version moso bamboo (*Phyllostachys edulis*) genome was designated as *PheGRF6a*. This naming was based merely on database annotations without systematic homologous sequence alignment. After careful re-evaluation during proofreading, we renamed this gene as PheGRF10 according to its closest ortholog in rice. This nomenclature principle is widely adopted in moso bamboo research, in which most bamboo genes are named after their rice orthologs. Since revisions were only marked in the PDF file during proofreading, our annotations may have failed to clearly convey our revision instructions to the editorial team. As a result, only partial instances of *PheGRF6a* were updated to *PheGRF10* in the published article, while other positions remained unchanged. Notably, *PheGRF6a* and *PheGRF10* represent the same moso bamboo gene (Ped09CXg23240).

The corrected **Figure 9** appears below.

**Figure 9:**
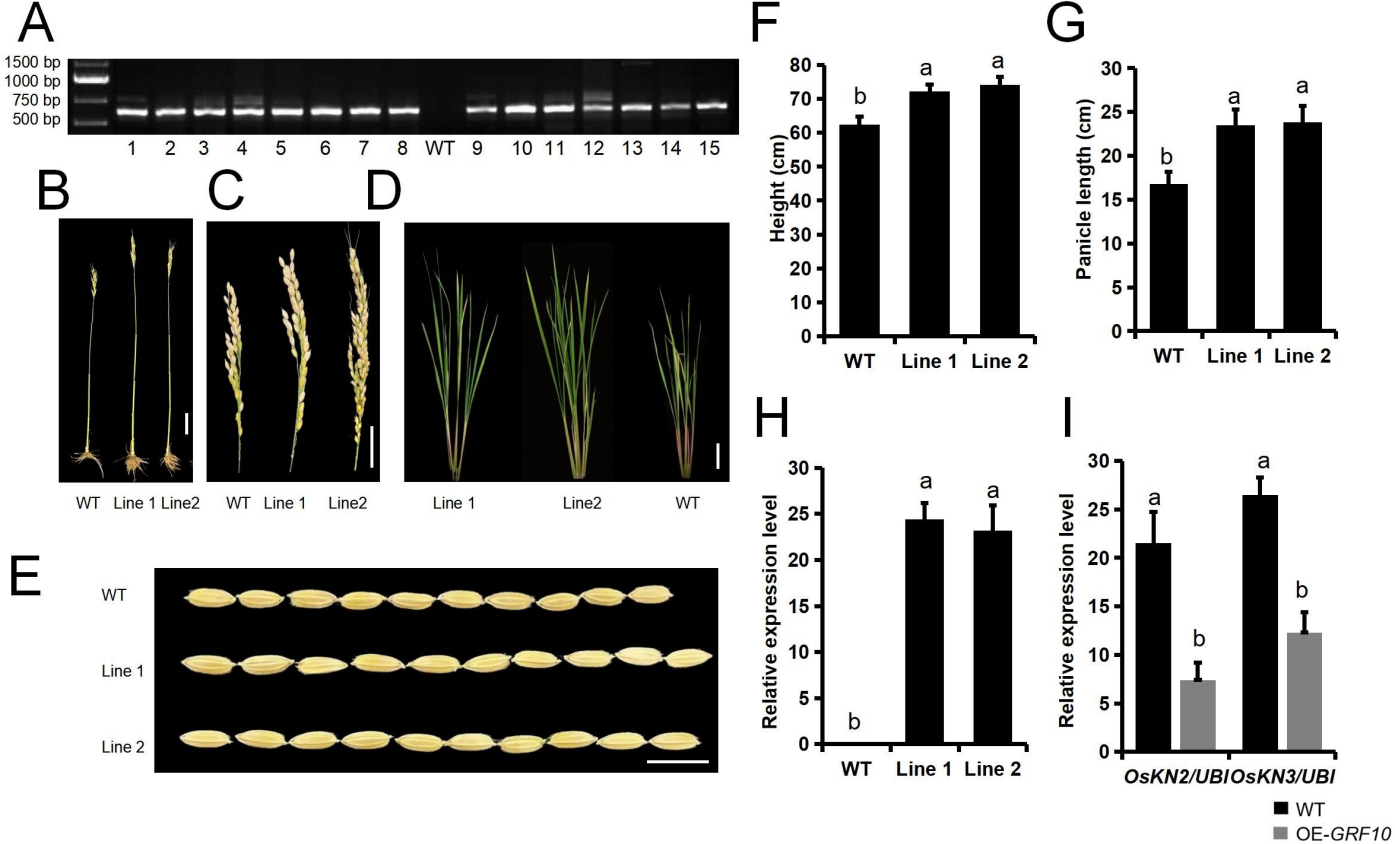
Phenotypic observation of *PheGRF10*-overexpressing rice lines. **(A)** Semi-quantitative PCR detection of the *PheGRF10*-overexpressing rice lines. **(B)** Observation of main stem phenotype in transgenic lines, scale bar, 10 cm. **(C)** Observation of panicle phenotype in transgenic lines, scale bar, 5 cm. **(D)** Observation of leaf phenotype in transgenic lines, scale bar, 10 cm, **(E)** Observation of seed length, 1 cm. **(F)** Statistics of plant height in overexpressing lines. **(G)** Statistics of panicle length in transgenic lines. **(H)** Expression level of *PheGRF10* in rice overexpression lines. **(I)** qRT-PCR analysis of downstream KNOX gene expression.

There was a mistake in the caption of **Figure 9** as published.

The gene name *PheGRF6a* is corrected to *PheGRF10*. The reason is the same as above. The corrected caption of **Figure 9** appears below.

“**Figure 9**. Phenotypic observation of *PheGRF10*-overexpressing rice lines. (A) Semi-quantitative PCR detection of the *PheGRF10*-overexpressing rice lines. (B) Observation of main stem phenotype in transgenic lines, scale bar, 10 cm. (C) Observation of panicle phenotype in transgenic lines, scale bar, 5 cm. (D) Observation of leaf phenotype in transgenic lines, scale bar, 10 cm, (E) Observation of seed length, 1 cm. (F) Statistics of plant height in overexpressing lines. (G) Statistics of panicle length in transgenic lines. (H) Expression level of *PheGRF10* in rice overexpression lines. (I) qRT-PCR analysis of downstream KNOX gene expression.”

There was a mistake in the caption of **Figure 10** as published.

The gene name *PheGRF6a* is corrected to *PheGRF10*. The reason is the same as above. The corrected caption of **Figure 10** appears below.

**Figure 10:**
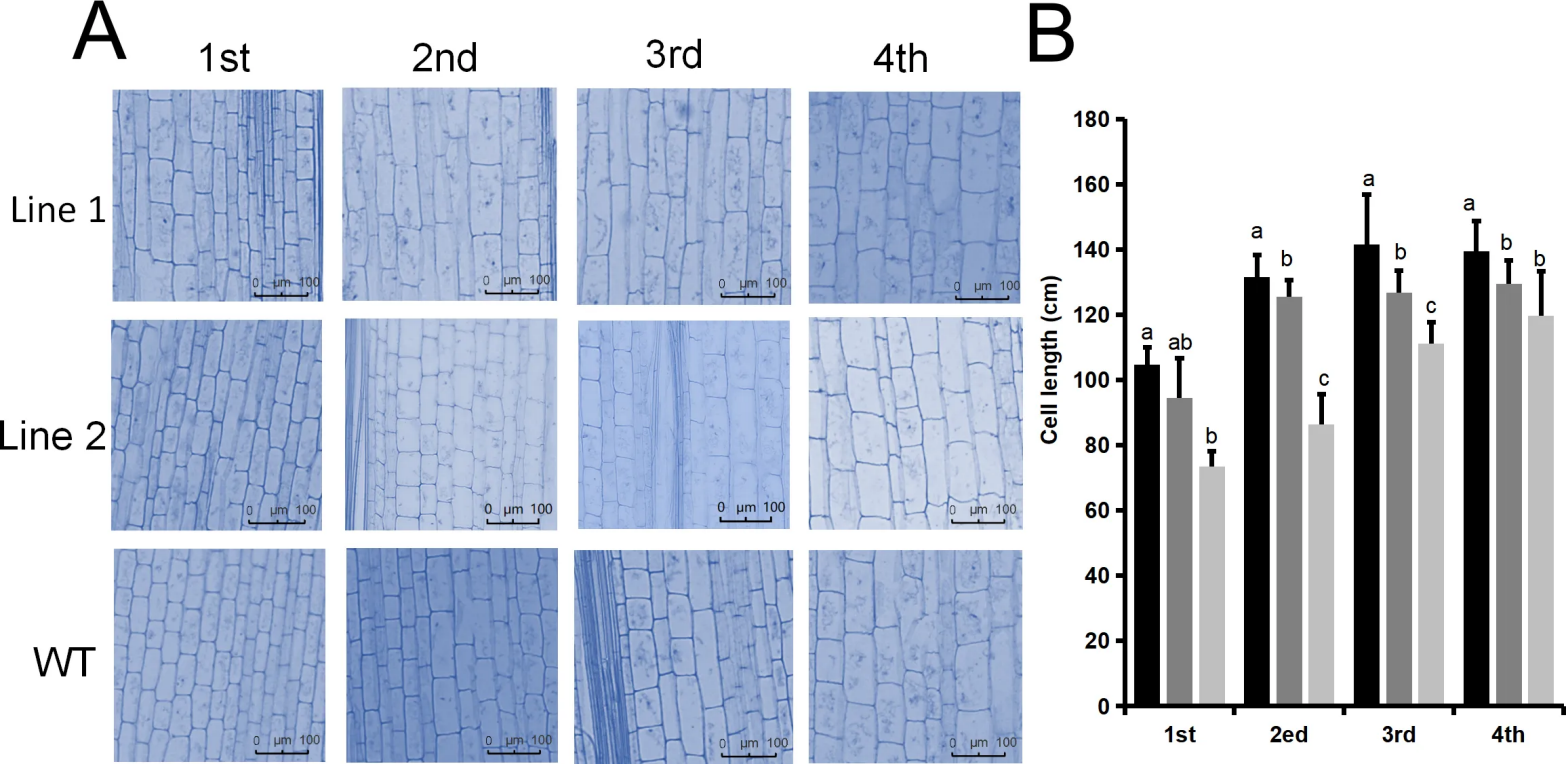
Observation of stem anatomical structures in *PheGRF10*-overexpressing rice lines. **(A)** Observation of stem anatomical structure and **(B)** Statistics of cell length. 1st, 2nd, 3rd, and 4th represent the 1st, 2nd, 3rd, and 4th internodes from the base upwards of rice stems.

“**Figure 10**. Observation of stem anatomical structures in PheGRF10-overexpressing rice lines. (A) Observation of stem anatomical structure and (B) Statistics of cell length. 1st, 2nd, 3rd, and 4th represent the 1st, 2nd, 3rd, and 4th internodes from the base upwards of rice stems.”

In the **Abstract**, The gene name *PheGRF6a* is corrected to *PheGRF10*. The reason is the same as above. The corrected Abstract is below:

“Internode length is an important trait of bamboo and a key indicator affecting the processing and utilization of bamboo materials. Shengyin bamboo is a dwarf variant of *Phyllostachys edulis* (Moso bamboo) with abnormally shortened internodes, yet its dwarfing mechanism has not been clarified. In this study, we adopted the method of Whole-Genome Bisulfite Sequencing (WGBS) for DNA methylation combined with RNA Sequencing (RNA-seq) to explore the key causes of dwarfism in Shengyin bamboo. Observations via paraffin sections and scanning electron microscopy (SEM) indicate that abnormal cell division and elongation in internodes are the key causes of dwarfism in Shengyin bamboo. Cell division-related genes such as GRF (Growth-regulating factor) and Cyclin are highly expressed during the cell division stage (early growth stage) of Moso bamboo internodes, while genes associated with cell elongation (Expansin-like A, EXPA) are highly expressed during the cell elongation stage (late growth stage) of Moso bamboo internodes. DNA methylation levels exhibit significant differences between Moso bamboo and Shengyin bamboo. Specifically, the DNA methylation level of Moso bamboo at the late stage of internode elongation is higher than that at the early stage, and this difference is significantly greater than the variation observed between the late and early stages of internode elongation in Shengyin bamboo. The expression of most genes shows a negative correlation with promoter methylation levels, indicating that methylation levels inhibit gene expression. Based on transcriptome data, *GRF10*, a gene potentially highly expressed in the early stage of internode growth of Moso bamboo under DNA methylation regulation, was screened out. Genetic transformation of rice showed that GRF10 can promote the growth and development of rice internode cells. In summary, under the regulation of DNA methylation, the expression of genes involved in internode cell division and elongation is inhibited, leading to fewer longitudinal cell lengths and cell numbers in the internodes of Shengyin bamboo compared to Moso bamboo, ultimately resulting in the shortened internodes of Shengyin bamboo.”

A correction has been made to the section **Material and Methods**, ***Overexpression of PheGRF10 (Ped09CXg23240)*,** page 4, paragraph 1. The gene name *PheGRF6a* is corrected to *PheGRF10* in the section ‘Overexpression of *PheGRF10* (Ped09CXg23240)’ in the methods. The reason is the same as above.

The text original stated:

“The full open reading frame of the *PheGRF6a* was amplified from cDNA using the primer pairs as follows: 5′- ATGGACCTGGGCGGGATGG-3 ′ (forward) and 5 ′- TCACACCAGGCGGATGCTCG-3′ (reverse). The amplified product was then cloned into the pMD18-T Vector. Subsequently, the PheGRF6a coding sequence was inserted into the Cambia1301:: PhePheGRF6a vectors, driven by the CaMV 35S promoter using the homologous recombination method. The recombinant vector was then introduced into rice (Oryza sativa L. subsp. japonica cv. Zhonghua 11) via Agrobacterium tumefaciens (EHA105)- mediated transformation following the method of Hiei et al. (1994). After extensive phenotypic characterization, PCR confirmations, and kanamycin selection, fifteen independent T3- generation transgenic lines were successfully established. The first, second, third, and fourth internodes from the base to the top of the stem in both transgenic lines and wild-type plants were collected as samples for paraffin sectioning. The middle portion of each internode was selected for section preparation, following the same methods described in Section 2.1.”

The corrected text is below:

“The full open reading frame of the *PheGRF10* was amplified from cDNA using the primer pairs as follows: 5′-ATGGACCTGGGCGGGATGG-3′ (forward) and 5′-TCACACCAGGCGGATGCTCG-3′ (reverse). The amplified product was then cloned into the pMD18-T Vector. Subsequently, the PheGRF10 coding sequence was inserted into the Cambia1301::*PhePheGRF10* vectors, driven by the CaMV 35S promoter using the homologous recombination method. The recombinant vector was then introduced into rice (*Oryza sativa* L. subsp. japonica cv. Zhonghua 11) via *Agrobacterium tumefaciens* (EHA105)-mediated transformation following the method of Hiei et al. (1994). After extensive phenotypic characterization, PCR confirmations, and kanamycin selection, fifteen independent T3-generation transgenic lines were successfully established. The first, second, third, and fourth internodes from the base to the top of the stem in both transgenic lines and wild-type plants were collected as samples for paraffin sectioning. The middle portion of each internode was selected for section preparation, following the same methods described in Section 2.1.”

The original version of this article has been updated.

